# NMR and LC–MS-based metabolomics to investigate the efficacy of a commercial bio stimulant for the treatment of wheat (Triticum aestivum)

**DOI:** 10.1007/s11306-024-02131-0

**Published:** 2024-05-21

**Authors:** Kamar Hamade, Ophelie Fliniaux, Jean-Xavier Fontaine, Roland Molinié, Laurent Petit, David Mathiron, Vivien Sarazin, Francois Mesnard

**Affiliations:** 1 UMRT INRAE 1158 BioEcoAgro, Laboratoire BIOPI, University of Picardie Jules Verne, 80000 Amiens, France; 2 AgroStation, Rue de La Station, 68700 Aspach-Le-Bas, France; 3 Plateforme Analytique, University of Picardie Jules Verne, 80000 Amiens, France

**Keywords:** *Wheat*, Bio stimulants, Metabolomics, Lodging resistance, Root growth and elongation, Cell wall lignification

## Abstract

**Introduction:**

Bio stimulants are substances and/or microorganisms that are used to improve plant growth and crop yields by modulating physiological processes and metabolism of plants. While research has primarily focused on the broad effects of bio stimulants in crops, understanding their cellular and molecular influences in plants, using metabolomic analysis, could elucidate their effectiveness and offer possibilities for fine-tuning their application. One such bio stimulant containing galacturonic acid as elicitor is used in agriculture to improve wheat vigor and strengthen resistance to lodging.

**Objective:**

However, whether a metabolic response is evolved by plants treated with this bio stimulant and the manner in which the latter might regulate plant metabolism have not been studied.

**Method:**

Therefore, the present study used ^1^H-NMR and LC–MS to assess changes in primary and secondary metabolites in the roots, stems, and leaves of wheat (*Triticum aestivum*) treated with the bio stimulant. Orthogonal partial least squares discriminant analysis effectively distinguished between treated and control samples, confirming a metabolic response to treatment in the roots, stems, and leaves of wheat.

**Results:**

Fold-change analysis indicated that treatment with the bio stimulation solution appeared to increase the levels of hydroxycinnamic acid amides, lignin, and flavonoid metabolism in different plant parts, potentially promoting root growth, implantation, and developmental cell wall maturation and lignification.

**Conclusion:**

These results demonstrate how non-targeted metabolomic approaches can be utilized to investigate and monitor the effects of new agroecological solutions based on systemic responses.

## Introduction

Fertilizers play a key role in meeting the world population’s food requirements by increasing crop yields and improving soil quality. However, the excessive use of unbalanced chemical fertilizers leads to severe environmental pollution that can affect human health and other natural resources, causing global economic losses (Choudhary & Kumar, 2019; Elhassani et al., 2019). In this context, the primary goal of sustainable agriculture nowadays is to provide promising and environmentally friendly strategies that could promote crop production and health, as well as enhance resilience in a changing climate (Colla & Rouphael, 2015). Over the last decade, research focusing on applying plant biostimulants has been steadily growing to partially replace chemical fertilizer use, aiming to improve agricultural practices and maintain crop yields in a more sustainable manner (Gupta et al., 2020).

Plant bio stimulants are a group of substances that, when applied to plants, enhance nutrition efficiency, protection against abiotic stresses, and crop productivity through the induction of natural biological processes such as cell division, root development and elongation, photosynthesis, dormancy and seeds germination …(du Jardin, 2015). Their use in high value-added agricultural production is wide-spread (wine production, vegetables…) (Calvo et al., 2014); However, their utilization in field crops remains limited, due to factors such as their cost, relatively limited research compared to traditional fertilizers and pesticides, a lack of understanding regarding their true effects, and their effectiveness, which can vary depending on several factors such as crop type, soil conditions, climate, and application methods.

It is important to note that the fine characterization of the impact of bio stimulants on phytohormone content and transcriptomes is not required for their commercialization. In most countries, bio stimulants are validated based on their effect on plant biomass under non-optimal mineral nutrition or abiotic stress conditions (Franzoni et al., 2022). Thus, farmers and users interrogate the real efficacy and effect of bio stimulants in real open-field conditions. Therefore, we aimed to clarify the usefulness of bio stimulants by investigating whether treated and non-treated plants can be distinguished after application.

Metabolites are the end products of the cellular regulatory pathways, and represent the processes that occur within biological systems in response to genetic factors and environmental changes (Nephali et al., 2021). Thus, applying metabolomic approaches would generate integrated information to explore the precise mechanisms of action through which bio stimulants exert their activity. In the present work, we selected a bio stimulant that was non-microbial, applied on leaves, registered for field crops and validated by several French agricultural cooperatives. We chose Agro-K®, a plant-based bio stimulant. For the cultivation of cereals such as wheat, rice, barley, and oats, this bio stimulant can be applied during the main vegetative growth stages of the crop in order to support root growth and implantation, leading to better soil anchoring (https://nufarm.com). While this bio stimulant is purported to have positive effects on wheat lodging, the response elicited by the plant has not been elucidated, nor has its exact mode of action on wheat been investigated. Herein, we explored the metabolic response of wheat plants treated with a bio stimulant, subjecting the roots, stems, and leaves of treated wheat plants to non-targeted nuclear magnetic resonance spectroscopy (^1^H-NMR) and liquid chromatography-mass spectrometry (LC-TOF–MS) analysis.

## Materials and method

### Agronomic field experiment

The trials were conducted on the experimental field of NORIAP located in Picardie, France (49°54′30.5ʺN 2°07′07.9ʺE), from March to May 2022. Winter wheat cultivar (*Triticum aestivum* L.) were grown following standard agronomical practices. The experiment consisted of two blocks: one control, which did not receive any treatment, and one that was treated with the bio stimulant. Each block was composed of ten rows with ten plants per row. We selected Agro-K®, a plant-based bio stimulant used to enhance lodging resistance for the trial. It is a complex mixture composed of potassium oxide (50%), phosphorus oxide (32%), amino acids (10%), and galacturonic acid (5%) extracted from the Nopal cactus. The bio stimulant Agro-K® was kindly provided by Nufarm (Colombes, France). Plants were treated with the bio stimulant according to the manufacturer’s instructions (https://nufarm.com): foliar spraying at a dose of 2 kg/ha when the crop was at the 1- to 2-node growth stage (BBCH29–BBCH31). Control and treated plants were harvested after 48 h of bio stimulant application, then every 7 days thereafter for a total of 7 time points (D2, D9, D16, D28, D35, D42, and D49). During harvest, the roots from each plant were carefully rinsed in water to remove soil and the plants were immediately placed into liquid nitrogen. Samples were stored at ‒80 °C and freeze-dried. The leaves, stems, and roots of each plant were then separately ground into a fine powder using a ball mill (Retsch MM200, Retsch Gmbh & Co, Germany). For each condition and time point, eight independent plants were randomly collected (n = 8) and subjected to further analysis. It should be noted that for stems, there were only 6 time points (D9, D16, D28, D35, D42 and D49) due to the lack of plant material during the early stages of stem development (D2 corresponds to the 1 cm ear stage).

### Metabolite extraction

The extraction method was carried out following the same protocol adapted in our previous study (Hamade et al., 2021). Briefly, 100 mg of powdered leaves, stems, and roots, were separately mixed with 800 µL water/methanol (1:1), and the samples were extracted for 10 min at 60 °C using a ThermoMixer® (Eppendorf AG, Hamburg, Germany) at 2000 rpm, followed by 30 min of sonication at 60 °C using an ultrasonic bath at 35 kHz. The mixtures were then centrifuged at 4 °C for 10 min at 12,000 × g. A volume of 400 µl of the supernatant was transferred into a 2 ml Eppendorf tube. The remaining residue was resuspended in 400 µl of extraction solvent water/methanol (1:1) and extracted a second time following the above-described procedure. A third extraction was carried out following the same extraction procedure. The supernatants were combined to obtain a final volume of 1.2 ml of which 800 µl were taken for NMR analysis and 400 µl for LC–MS analysis.

### Metabolite analysis by NMR

#### Sample preparation

Sample pH was adjusted to 6.00 ± 0.02 for NMR analysis. Samples were dried in a Speed vacuum concentrator and then resuspended in 800 µl of a deuterated mixture of methanol-d4:KH_2_PO_4_ buffer (0.1 M) in D_2_O (1:1) at pH 6.0 with 0.0125% TMSP and NaN_3_ (0.6 mg/ml). Then, samples were vortexed, submitted to an ultrasonic bath, and centrifuged to obtain a clear supernatant which was transferred into 5 mm NMR tubes for further analysis.

#### NMR data acquisition and processing

The NMR protocol for untargeted metabolomics was adapted from our previous study (Hamade et al., 2021). NMR experiments were performed with a Bruker Avance III 600 MHz spectrometer (600.13 MHz for ^1^H and 150.91 MHz for ^13^C), with a 5 mm multinuclear detection broadband, equipped with z-gradient (TXI 5 mm tube). For quantitative analysis, classical 1D ^1^H-NMR spectra were collected at 131 K data points using 128 scans and a spectral width of 8417 Hz with a relaxation delay of 25 s. For metabolomic profiling, a NOESY-1D water suppression pulse sequence was used and generated spectra were collected at 131 K data points using 256 scans, and a spectral width of 8417 Hz with a relaxation delay of 25 s. The ^1^H-NMR data obtained were automatically phased, baseline corrected and calibrated by the internal reference (TMSP) signal at 0.0 ppm using the software Topspin (version 3.5) from Bruker (Deborde et al., 2019). Two-dimensional J-resolved spectra (J-RES) were recorded with a 2 s relaxation delay, using 16 scans per 64 increments accumulated into 64 K data points, with spectral widths of 8417.5 Hz in the F2 dimension and 50 Hz in the F1 dimension. Two-dimensional ^1^H-^13^C HSQC spectra were processed with a 2 s relaxation delay, using 64 scans per 256 increments that were accumulated into 4 K data points, with spectral widths of 8417.5 Hz in the F2 dimension and 50 Hz in the F1 dimension. Each of the 2D acquisitions were carried out by analyzing one control and one treated sample of each plant part at each harvest time point. 1D NOESY spectra were converted into an ASCII file format, using TopSpin 3.2 software and the data from each plant part (leaves, stems, and roots) were imported separately into MATLAB software (version 2017b, Mathworks Inc, Natick, MA, USA) for baseline correction with the package airPLS 2.0 (Gan et al., 2006). All spectra were aligned simultaneously using the icoshift algorithm (v 1.2.1) with manually defined alignment bins. Then, the “dynamic adaptive binning (DAB)” algorithm (Anderson et al., 2011) was applied for automatic spectra bucketing and bin integrations. The regions for methanol-d4 (3.34–3.30 ppm), unsuppressed water (4.85–4.70 ppm), and TMSP (0.01–0 ppm) were removed. The resulting data set corresponding to the bin area was then used for statistical analysis. For the identification of metabolites, a comparison was performed between experimental 1D and 2D NMR spectra with the spectra and chemical shifts of reference compounds available in the database developed in our laboratory.

### Metabolite analysis by LC–MS

#### Sample preparation

Extracts of leaves, stems, and roots were diluted 60, 40, and 10 times, respectively, with methanol/water (50/50). All samples were filtered through 0.22 µm PTFE syringe filters and placed in glass vials prior to LC–MS analysis. A blank sample, containing no extract, as well as a quality control (QC) sample were analyzed for quality assessment. The QC sample, prepared by mixing 10 µL from each test sample, was injected after every ten samples to confirm the stability and reproducibility of the analysis. The samples of leaves, stems, and roots were analyzed in a random run order.

#### LC–MS data acquisition and processing

UPLC-HRMS analysis was performed using an ACQUITY UPLC I-class chain coupled to a Vion IMS Q-TOF high resolution mass spectrometer, equipped with an electrospray (ESI) (Waters, Manchester, UK) ionization source (Z-spray) and an additional spray for the reference compound (Lock Spray). Chromatographic separation was performed using a Kinetex C18 column (1.7 µm, 100 × 2.1 mm, Phenomenex, Torrance, CA, USA). The column temperature was maintained at 50 °C. The flow rate was set at 0.5 ml/min and the injection volume was 1 µl. The mobile phases used were A: H2O and B: methanol, both with 0.01% formic acid. The gradient changed from 5% B at t = 0 min to 95% B at t = 7 min, holding for 2 min, and at t = 9 min B decreased to 5% and was held for 2 min; the total run time was 11 min. Both positive and negative ionization modes were performed by ESI mass spectrometry and a PDA diode array detector (UV detection between 190–500 nm). The ESI source was operating using the following conditions: cone voltage, 120 V; source offset, 20 V; source temperature, 120 °C; desolvation gas temperature, 450 °C; desolvation gas flow, 800 L/h; and cone gas flow, 50 L/h. Nitrogen (> 99.5%) was employed as the desolvation gas. Time-of-flight (TOF) MS was operated in sensitive mode. The data were acquired in high-definition MSE (HDMSE) over a mass range of *m*/*z* 50–2000 Da, at a mass resolving power of 50,000 FWHM and a scan time of 0.2 s. The obtained spectral data were acquired and processed using UNIFI software (version 1.9.4, Waters), allowing the generation of an untargeted metabolomics data matrix, including retention time and m/z, sample names, and ion intensities for each sample set. Then, these matrices were cleaned by removing the metabolic features with relative standard deviation (% RSD) greater than 35% in the QC and the metabolic features present in the blank. A minimum intensity threshold was chosen to keep the metabolic features. Some compounds were identified by matching the accurate masses, retention times and fragment pattern with those of the reference standards, while others were annotated with exact masses of molecules available in databases, including the KnaPSacK metabolome database, MassBank, Kyoto Encyclopedia of Genes and Genomes and MetFrag, as well as the UNIFI library. This identification was also confirmed using previous publication on the same molecules (Tais et al., 2021).

### Statistical analysis

Multivariate statistical analysis was performed, using MATLAB software. For each of the three plant parts, the obtained datasets for NMR and LC–MS were fused and subjected to a multi-block orthogonal partial least squares discriminant analysis (Multiblock OPLS-DA) in order to explore the total variation of the data and study the classification of the control and treated samples. The signals of the discriminating metabolites were identified using loading plots. The Wilcoxon Rank Sum test was used in R’s statistics base-package in order to test the significant differences in metabolite content between analyzed groups with different p-values (p < 0.05, p < 0.01, and p < 0.001).

## Results and discussion

**Fig. 1:**
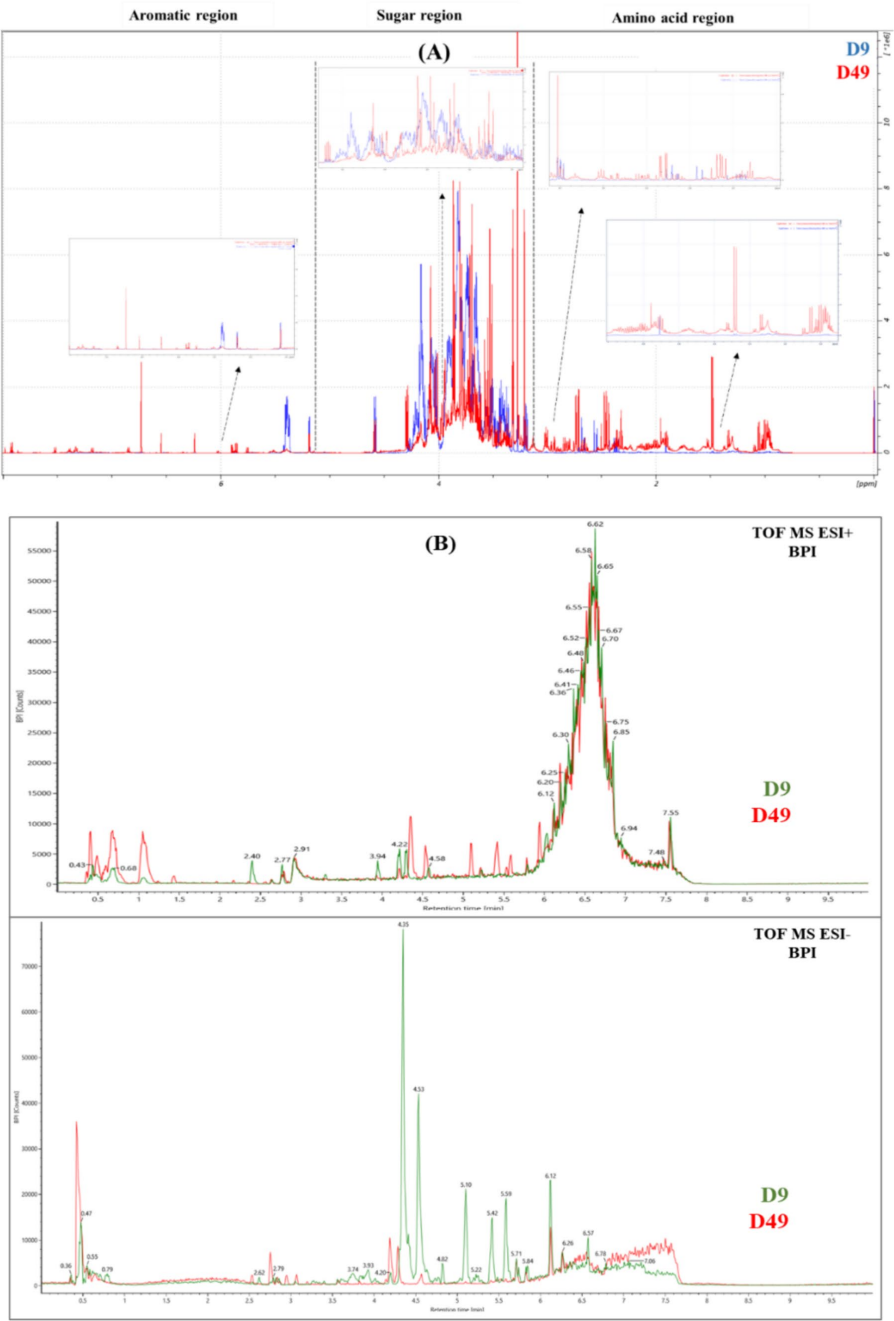
Comparison of.^1^H NMR spectra (600 MHz, in MeOD/D_2_O) (**A**) and LC-Q-TOF/MS BPI chromatograms in positive (top) and negative (bottom) ion modes (**B**), resulting from stem samples of control plants harvested at two different time points (D9 and D49)

To study the impact of the bio stimulant treatment on the wheat metabolome, we compared the metabolite composition of each of the three parts (roots, stems, and leaves) of control and treated plants at different time points. The LC–MS and NMR spectral data obtained for each of the three plant parts were subjected separately to a multivariate non-targeted analysis. For each of the plant parts, especially for the stems and leaves, NMR as well as LC–MS spectra showed a difference in the metabolic profile between samples harvested at different time points. The greatest difference was observed between samples harvested at D2, D9, and D16 and those harvested at D28, D35, D42, and D49. In this regard, NMR and LC–MS data collected for the two sample groups were processed separately. An example of the superposition of two ^1^H NMR spectra (A) and two LC–MS chromatograms (B) of stem samples selected from the two different groups is shown in Fig. 1**.**

**Fig. 2:**
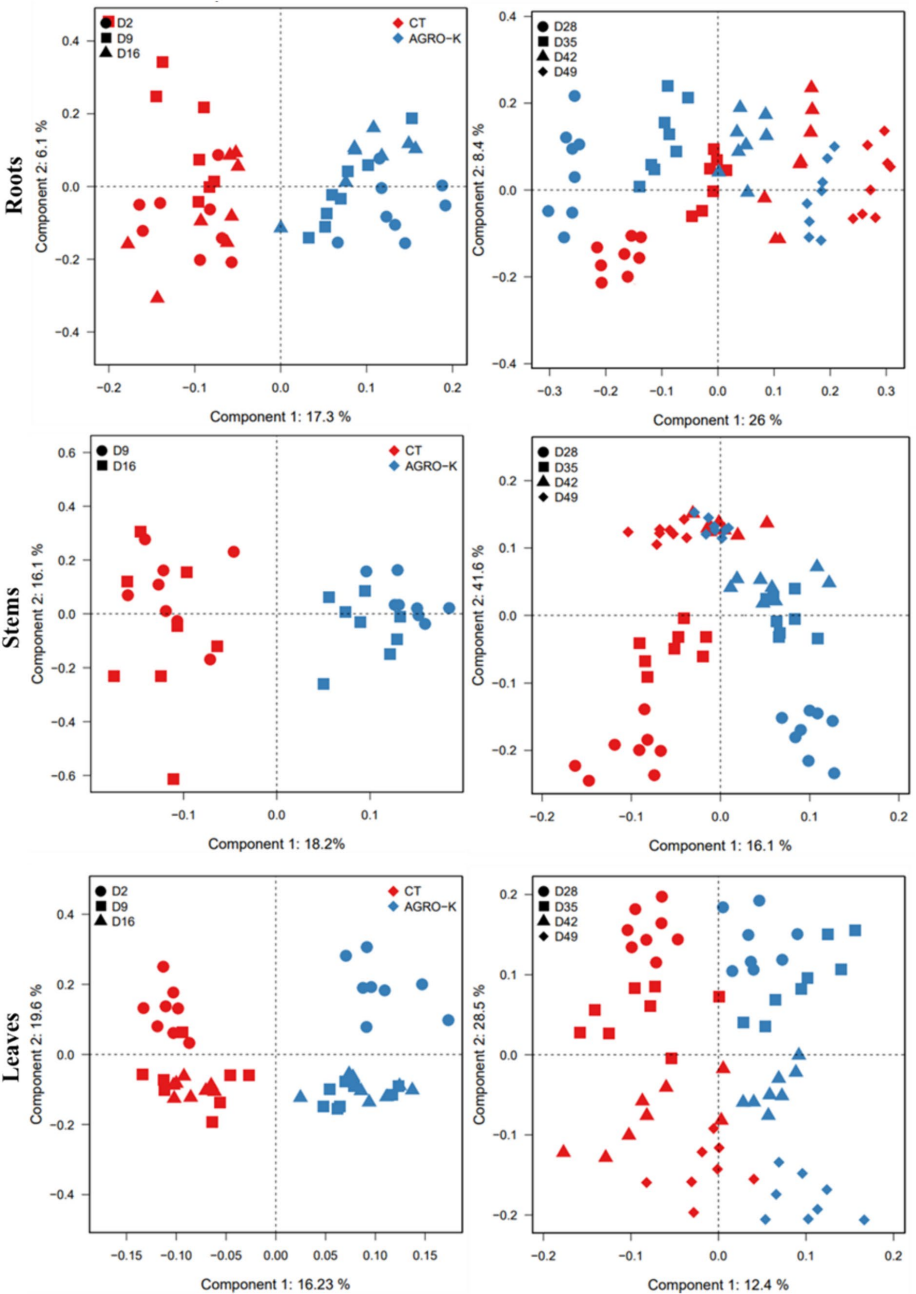
Score plot of multi-block orthogonal partial least squares discriminant analysis (OPLS-DA) of metabolite content in wheat roots, stems, and leaves based on.^1^H-NMR and LC–MS data (Pos/Neg ion mode), for control (Red) and treated (Blue) samples, at different time points: D2, D9, D16, D28, D35, D42, D49

The OPLS-DA, shown in Fig. 2, exhibited good clustering of control and treated samples of the various plant parts according to harvest day, strongly indicating the distinctness of the metabolomes of the root, stem, and leaf samples at different stages of growth. These metabolic variations represent the impact of growth occurring in wheat over the 49-day study period. Thus, D2, D9, D16, D28, D35, D42, and D49 samples could be considered as separate groups for each of the three organs.

**Fig. 3:**
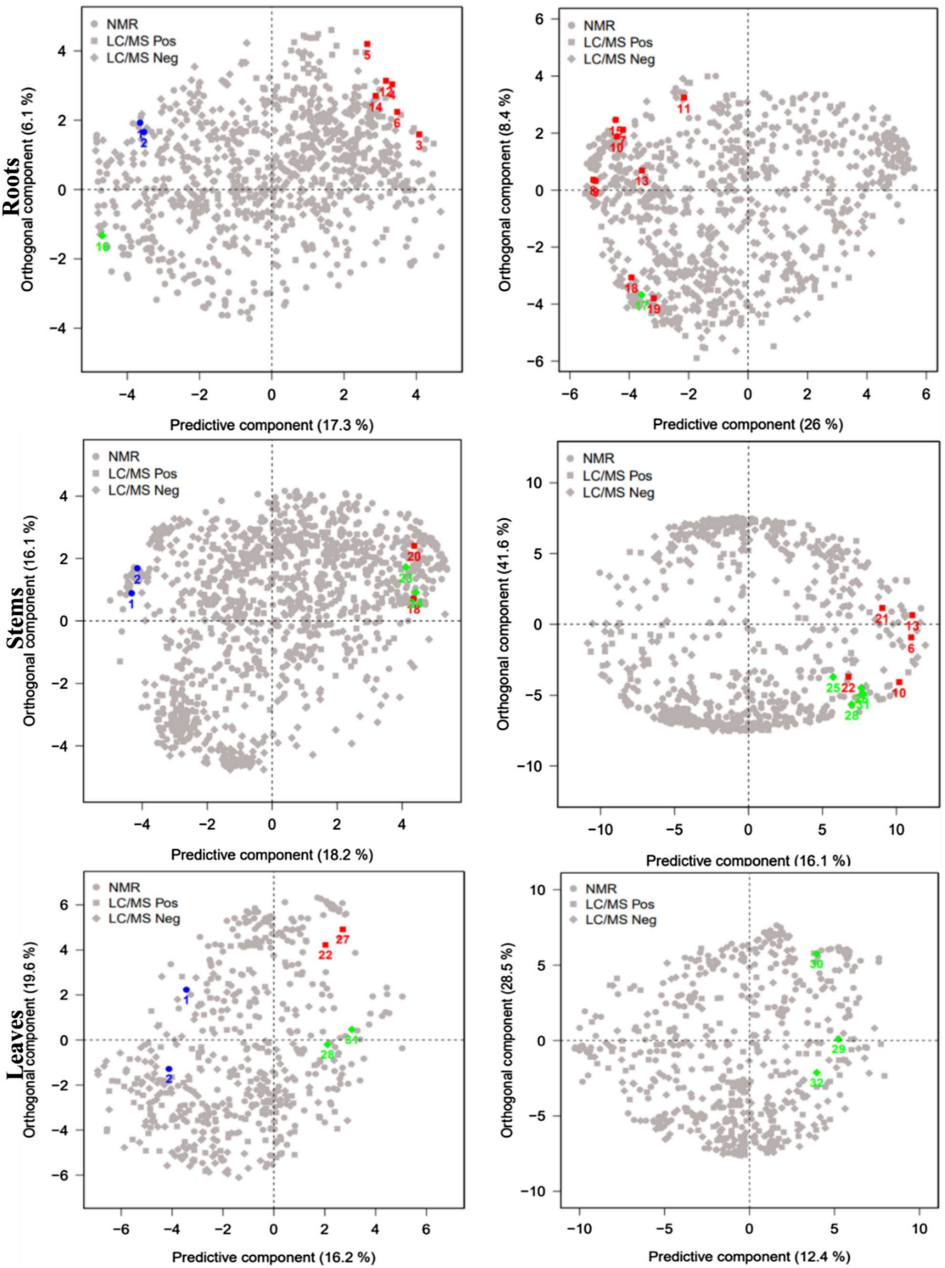
OPLS-DA loading plot, showing the distribution of metabolites responsible for the discrimination of the corresponding score plot (Fig. 2), of wheat roots, stems and leaves, in control and treated conditions**,** at different time-points: D2, D9, D16, D28, D35, D42, D49. • discriminant variables detected with NMR; ▪ detected with LC–MS in positive ionization; ⬪ detected with LC–MS in negative ionization. **(1)** Arginine; **(2)** Lysine; **(3)** N-feruloyl-agmatine; **(4)** N-caffeoyl-agmatine; **(5)** N-5-hydroxyferuloyl-agmatine; **(6)** N-coumaroyl-agmatine; **(7)** N-feruloyl-putrescine; **(8)** N-coumaroyl-hydroxy-putrescine; **(9)** N,N-Bis-coumaroyl-putrescine; **(10)** N-coumaroyl-putrescine; **(11)** N,N-Bis-feruloyl-putrescine; **(12)** N-caffeoyl-cadaverine; **(13)** N,N-coumaroyl-caffeoyl-cadaverine; **(14)** C3H6O3-O-feruloyl; **(15)** C6H12O3-O-feruloyl; **(16)** S(8–8)G(4-O-8)G; **(17)** G(5–8)S(4-O-8)G; **(18)** S(8–8)S(4-O-8)G: **(19)** G(8-O-4)S(8–8)S(O-4–8)G; **(20)** N-coumaroyl-hydroxy-agmatine; **(21)** N-feruloyl-hydroxy-putrescine; **(22)** C5H10O2-O-feruloyl; **(23)** S(8_8)S(4-O-8)S; **(24)** Quercitin -3ʹ-O-rutinoside; **(25)** kaempferol O-hexoside-O-deoxy-hexoside; **(26)** Apigenin C-pentoside-C-hexoside; **(27)** C7H16O3-O-feruloyl; **(28)** Apigenin C-pentoside-C-pentoside; **(29)** Apigenin C-pentoside-C-hexoside-O-(5-hydroxy-feruroyl); **(30)** Apigenin C-pentoside-C-hexoside-O-hexoside; **(31)** Apigenin C-pentoside-C-hexoside-O-feruloyl; **(32)** Apigenin C-pentoside-C-hexoside-O-sinapoyl

In addition, at each stage of growth, the OPLS-DA score plot of the roots, stems, and leaves consistently revealed distinct clustering of control samples apart from the treated samples. These results highlight the differences in metabolic composition between the control and treated samples in the various plant parts. This observation suggests that the bio stimulant treatment induced a metabolic response that persisted throughout all the studied growth stages in the roots, stems, and leaves of wheat. The next step to understand the mechanisms of action of the bio stimulant consisted of identifying the major changes in metabolic content of each plant part after being treated. The corresponding loading plots of the OPLS-DA obtained from NMR and LC–MS data (Fig. 3**)**, showed the distribution of metabolites in control and treated samples. The identification of the metabolites most responsible for the separation between control and treated samples indicate that the change is not restricted to a specific chemical classes, but involves different metabolic pathways, in the different plant parts.

**Fig. 4:**
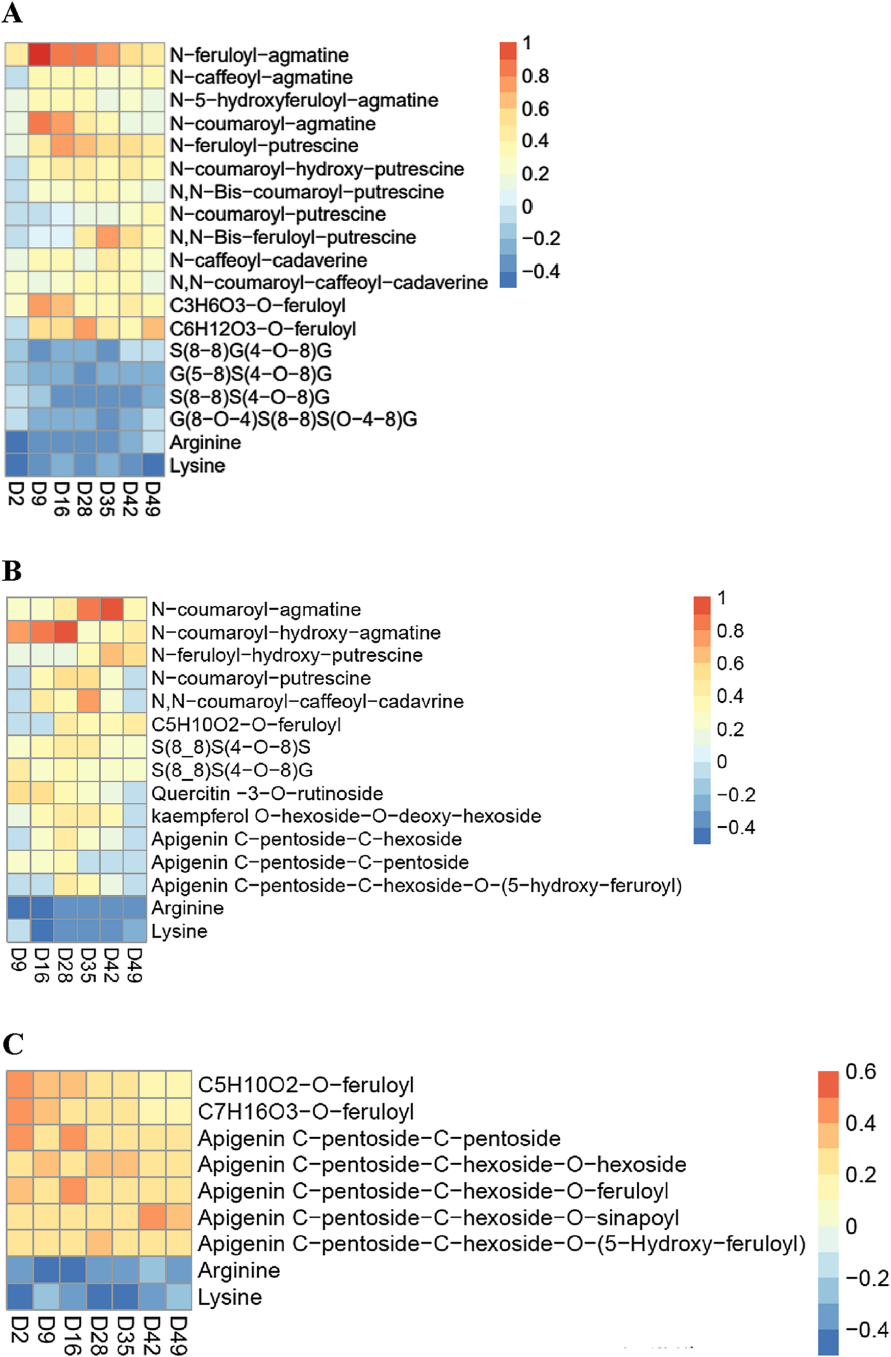
Comparison of major metabolite content reorganization in the roots (**A**), stems (**B**), and leaves (**C**) of wheat after treatment with the bio stimulant at different time points: D2, D9, D16, D28, D35, D42, and D49. Each color represents a specific value of the mean relative response ratio T/C (content in plant treated by the bio stimulant/content in control plant), on a logarithmic scale (log10). Negative values represent a lower content and positive values a higher content of metabolites in treated plants compared to those in the control

Figure 4 provides a heat map representing the log tenfold changes of the metabolites that contribute the most to the discrimination of the control and treated samples, in the corresponding score plot OPLS-DA of the different plant parts.

In the roots and stems, the major change observed was in the amount of hydroxycinnamic acid amides (HCAAs), which are polymers made from hydroxycinnamic acids (HCAs) and polyamines (PAs). In the roots, a total of 11 compounds involved in the HCAA metabolic pathway were identified as significantly more abundant in the treated samples than in the control samples at different growth stages. The most notable increase in HCAA content was observed for N-feruloyl agmatine. This compound showed a rapid transient increase in content after two days of treatment, and its content reached the highest increase with a T/C ratio (calculated by the formula: T/C = content in treated plant /content in control plant) of 10.5, 7.2, and 7.1 at D9, D16, and D28, respectively. A 5.43-, 3.88-, and 2.61-fold increase in the content of this metabolite was also observed in the roots of treated samples at D35, D42, and D49, respectively. Other HCAs-agmatine conjugates accumulated in the same way in the roots when the plants were treated. The levels of *N*-caffeoyl-agmatine, *N*-5-hydroxy-feruloyl-agmatine, and *N*-coumaroyl-agmatine increased at different harvest time points. The amount of *N*-feruloyl-putrescine was shown to be 1.49, 2.85, 6.18, 4.78, 3.84, 3.61, and 2.58 times higher in the treated roots than those in the control at D2, D9, D16, D28, D35, D42, and D49, respectively. There was also a significant increase in the amount of HCAs-putrescine conjugates such as *N*-coumaroyl-hydroxy-putrescine, *N,N*-bis-coumaroyl-putrescine, *N*-coumaroyl-putrescine, and *N,N*-bis-feruloyl-putrescine in the treated samples compared to those in the control samples at different growth stages.

In addition, an up-accumulation of HCAs-cadaverine conjugates such as *N*-caffeoyl-cadaverine and *N,N*-coumaroyl-caffeoyl-cadaverine was observed in the roots of the treated samples compared with those of the control samples at different time points. Almost similar trends in HCAA content were observed in the stems as previously seen in the roots after treatment with the bio stimulant. The levels of five HCAAs increased significantly in the stems of treated samples when compared to those of control samples. Of these, *N*-coumaroyl-agmatine and *N*-coumaroyl-hydroxy-agmatine showed the highest increase, with a rise of 8.4-fold at D28 and 9.54-fold at D42 post-treatment, respectively. As for *N*-feruloyl-hydroxy-putrescine, *N*-coumaroyl-putrescine, and *N,N*-coumaroyl-caffeoyl-cadaverine, they were accumulated in different levels in the treated samples at D28, D35, D42, and D49. These results indicate that the HCAA pathway was strongly induced in wheat after treatment with the bio stimulant. The reorganization, appearing two days post-treatment and becoming more visible over the harvest period, indicated that the bio stimulant had a continuous and long-term impact (49 days) on the biosynthesis of HCAAs in wheat plants.

Interestingly, a remarkable decrease in the content of amino acids, such as arginine, was observed in the roots, stems, and leaves after treatment with a T/C ratio ≤ 0.5 at the different stages of growth. A similar decrease was also observed for lysine in the different plant parts and for phenylalanine in the stems and roots. As mentioned earlier, HCAAs are formed through the conjugation of aliphatic PAs, such as putrescine, agmatine, and cadaverine, with hydroxycinnamic acids, such as p-coumaric, ferulic, and caffeic acids, along with some glycosylated forms.

**Fig. 5:**
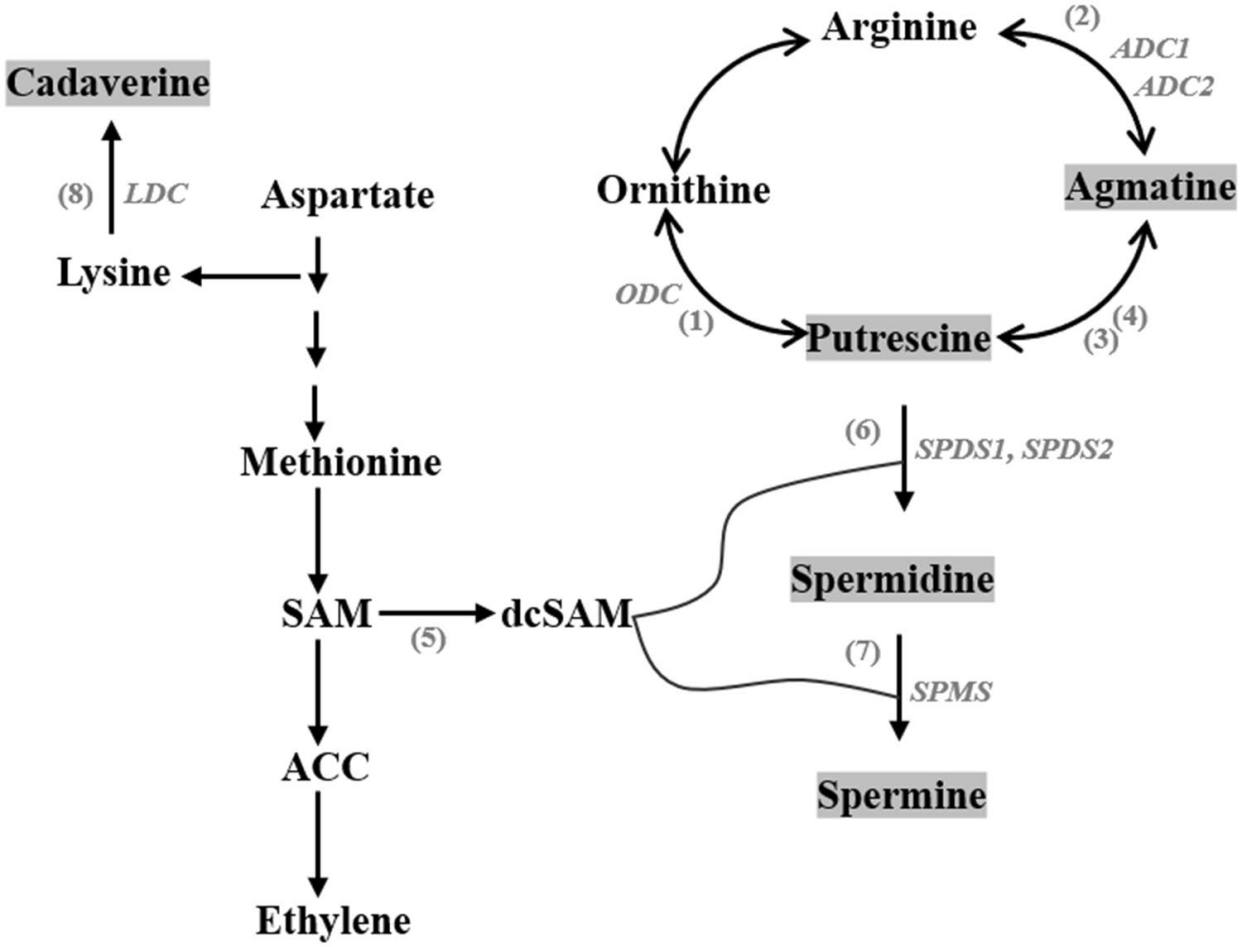
Biosynthesis pathways of main plant polyamines (Put, Agm, Spd, Spm, and Cad). Based on Gill and Tuteja (2010). Enzymes involved: (1) ODC, ornithine decarboxylase; (2) ADC, arginine decarboxylase; (3) agmatine iminohydrolase; (4) N-carbamoylputrescine amidohydrolase; (5) SAMDC, SAM decarboxylase; (6) SPDS, spermidine synthase; (7) SPMS, spermine synthase; (8) LDC, lysine decarboxylase; ACC, 1-aminocyclopropane-1-carboxylic acid; dcSAM, decarboxylated S-adenosylmethionine

In plants, the diamine putrescine is generally synthesized by two pathways. One pathway starts with the decarboxylation of arginine to give agmatine via arginine decarboxylase (ADC) followed by additional steps to produce putrescine. The other starts with the decarboxylation of ornithine to directly give putrescine via ornithine decarboxylase (ODC). The diamine cadaverine is derived from the amino acid lysine via lysine decarboxylase (LDC) (Fig. 5) (Gill & Tuteja, 2010).

Therefore, the accumulation of HCAAs in the treated samples provides an explanation for the simultaneous decrease in the contents of arginine and lysine, which are direct precursors of PAs, essential for production of HCAAs. Hydroxycinnamic acids are end products of the phenylpropanoid pathway, which begins with the deamination of phenylalanine by phenylalanine ammonia lyase. Thus, the decrease in phenylalanine content observed in the treated samples could be explained by the redirection of the cell to induce deamination of this compound in order to yield more hydroxycinnamic acid, which is involved in the biosynthesis of HCAAs.

An accumulation of HCA conjugates, specifically ferulic acid conjugates, was also observed in samples after treatment with the bio stimulant. This was the case of C_3_H_6_O_3_-O-feruloyl in the roots and leaves, C_5_H_10_O_2_-O-feruloyl in the leaves and stems, C_6_H_12_O_3_-O-feruloyl in the roots, and C_7_H_16_O_3_-O-feruloyl in the leaves. This accumulation was also in accordance with the increase of HCAAs. Thus, it is likely that hydroxycinnamic acid accumulates in different conjugated forms, that might serve as precursors in HCAA production.

HCAAs are purported to play an important role in a range of plant growth and development processes including flower development, pollen wall formation, cell division, and biotic and abiotic stress responses (Facchini et al., 2002; Luo et al., 2009). As final products of polyamine catabolism or as polyamine or phenolic storage forms, HCAAs seem to play an important role in primary root growth as well as during lateral and adventitious root formation (Couée et al., 2004). Studies in several plant species have shown that variations in the endogenous levels of free, conjugated, or macromolecule-bound PAs, by gene manipulation or by exogenous treatment, can have a drastic impact on root development and its subsequent architecture. For example, in *Hyoscyamus musticus*, endogenous depletion of agmatine, spermidine, and spermine levels resulting from decreased activity of arginine decarboxylase led to decreased root elongation in lateral roots in hairy root cultures (Biondi et al., 1993). On the other hand, Tarenghi & Martin-Tanguy (1995) showed that in (*Fragaria* x *ananassa* Duch.) micro cuttings, exogenous treatment with putrescine led to increased endogenous putrescine levels, greater number of adventitious roots and increased root length (Tarenghi & Martin-Tanguy, 1995). Similarly, in *Pringlea antiscorbutica*, a positive correlation between endogenous agmatine content and growth rate of the primary root was also found (Hummel et al., 2002). Another study showed that PAs can promote root growth and elongation by increasing cell division and differentiation in *Pinus virginiana* (Tang & Newton, 2005). For wheat, the application of exogenous PAs has been shown to improve tolerance to drought conditions by enhancing the molecular and metabolic pathways of PAs (Ebeed et al., 2017). The exact mechanisms of action of PAs remain controversial, but several studies have confirmed that PAs interact in synergistic or antagonistic ways with various plant hormones such as abscisic acid, salicylic acid, auxins, ethylene, and gibberellic acid to regulate growth and development of plants (Gonzalez et al., 2011; Kolotilin et al., 2011; Mendes et al., 2011; Takács et al., 2016). Furthermore, HCAAs have been proven to increase cell wall thickness in order to protect plants against pathogens. PAs bind to HCA phenolic resins, leading to the formation of HCAA molecules that have the ability to deposit into cell walls (Zeiss et al., 2021). HCAAs in the cytosol may be transported to different vesicles and then to the plasma membrane, allowing the deposition of HCAAs into the cell wall. Glutathione S-transferase may act as an amide carrier protein for HCAA translocation to the plasma membrane (Macoy et al., 2015). Moreover, HCAAs, such as coumaroyl putrescine, feruloyl putrescine, cis-*p*-coumaroyl agmatine, and feruloyl agmatine, were found to increase cell wall thickness in wheat (Gunnaiah et al., 2012). HCAAs constitute the polyaromatic domains of suberin which increased the thickness of cell walls and limited the spread of pathogens (Graça, 2010). It has also been demonstrated that HCAAs can enhance plant cell walls by inducing the synthesis of lignin and deposition of the glucan polymer callose (Macoy et al., 2022). Lodging is defined as the displacement of the plant shoot from its vertical position and can be divided into stem lodging and root lodging. While stem lodging refers to the buckling of the stalk/stem to the ground, root lodging is the failure of the roots to provide anchorage (Guo et al., 2021). Lodging resistance is related to the morphological and anatomical traits of the root and stem, and influenced by their chemical composition (Shah et al., 2019). Based on the above, it may be assumed that treatment with the bio stimulant can improve wheat resistance to lodging by promoting root development and soil anchorage, and ensuring cell wall rigidity of stems and roots. This is inferred from the increased abundance of HCAAs in the roots and stems treated with the bio stimulant, which have been shown by various studies to be involved in root growth and development as well as in cell wall formation.

Moreover, our results showed that oligolignol levels were altered in the wheat roots and stems after the application of the bio stimulant. In the roots, a significant decrease in the amounts of S(8–8)G(4–O–8)G, G(5–8)S(4–O–8)G, S(8–8)S(4–O–8)G, and G(8–O–4)S(8–8)S(O–4–8)G was observed in treated samples when compared to those in the control samples. Conversely, in the stems, an accumulation of S(8–8)S(4–O–8)S and S(8–8)S(4–O–8)G was observed in treated samples compared to the control samples. The aforementioned oligolignols are composed of coniferyl (G) and sinapyl (S) alcohol monomers, which are connected via 8–O–4 (β-aryl ether type), 8–5 (phenylcoumaran type), and 8–8 (resinol type) linkage motifs. These oligolignols are formed during the oxidative step of lignin polymerization (Morreel et al., 2010). In fact, lignin is an aromatic heteropolymer that is progressively assembled in the plant cell wall by an oxidative polymerization process in which coniferyl alcohol, sinapyl alcohol and *p*-coumaroyl alcohol are first enzymatically oxidized by peroxidase and/or laccases to form guaiacyl (G), syringyl (S), and *p*-hydroxyphenyl (H) radicals, respectively. These monolignol radicals then undergo spontaneous polymerization (Simon et al., 2017). The combinatorial coupling of monolignol radicals results in highly variable racemic lignin polymers with different physicochemical features (Mottiar et al., 2016). In wheat, lignin is predominantly composed of guaiacyl (G) and syringyl (S) units that are derived from combinatorial radical–radical coupling of coniferyl and sinapyl alcohol, respectively (Ralph et al., 2004). Lignin is mainly present in secondary-thickened cell walls, allowing the transport of water and nutrients, providing structural support and giving rigidity and strength to stems to stand upright (Yi Chou et al., 2018). Several studies have demonstrated a significant relationship between the lignin content in wheat stems and roots and lodging resistance (Li et al., 2021). The accumulation of oligolignols in treated stems, compared to the control, suggests that treatment with the bio stimulant may enhance stem lignification by inducing the oxidative polymerization of monolignols. Conversely, a decrease in oligolignol content was observed in the roots after treatment. This led to the hypothesis that these compounds may be synthesized in the root tissue and then transported to the stem tissue to supplement the lignification process in stems, thereby enhancing stem thickness, wall thickness, and reducing the risk of lodging. This hypothesis is supported by previous research suggesting that cells can export lignin precursors into shared walls of neighboring cells (Hosokawa et al., 2001; Pandey et al., 2015; Pesquet et al., 2013; Smith et al., 2017; Tobimatsu et al., 2013). The mechanisms underlying the export of lignin precursors to the cell walls of lignifying neighbors remain unclear. This export could be facilitated by active transporters, diffusion through the cell membrane, or diffusion facilitated by channels in the membrane.

Galacturonic acid is far known to act as an plant immune system elicitor (Hahn et al., 1981; Jin & West, 1984). It functions as Damage Associated Molecular Pattern (DAMP) (Hou et al., 2019), whose can be recognized by Pattern Recognition Receptor (PRR; ref) and so be used for plant bio stimulation (Krzyzaniak et al., 2018; Quintana-Rodriguez et al., 2018). Recent studies on various plant models have demonstrated that treatment with exogenous Galacturonic Acid Oligosaccharides as an elicitor leads to the accumulation of transcripts such as the phenylalanine ammonia-lyase gene (PAL). PAL is the first enzyme in the phenylpropanoid pathway, which is crucial for the production of phytoalexins and lignin. Additionally, treatment with this compound stimulates changes in several defense genes, including salicylic acid (SA), which plays a role in various physiological responses, including the strengthening of cell walls by increasing lignin and callose production. (Butselaar & Ackerveken, 2020; Ochoa-Meza et al., 2021). Therefore, we can hypothesize that galacturonic acid present in the bio stimulant formulation, act as an elicitor to induce the accumulation of HCCAS and lignin production in wheat, through the stimulation of salicylic acid pathway, since this latter is known to be involved in the regulation of polyamine and lignin biosynthesis (Canales et al., 2019).

A significant change in the content of metabolites involved in the flavonoid biosynthetic pathway was also observed in the stems and leaves of the treated samples compared to the control samples. In the stems, a total of five flavonoids were identified as being accumulated in plants treated with the bio stimulant at D9, D16, D28, D35, and D42, but no significant accumulation was detected at D49. The highest accumulation was observed for flavonol di-glycosides, including quercetin 3’-O-rutinoside and kaempferol O-hexoside-O-deoxy-hexoside, that reached an increase in content with a T/C ratio of 3.65 at D9 and 2.65 at D35, respectively. Flavone di-glycosides such as apigenin C-pentoside-C-hexoside and apigenin C-pentoside-C-pentoside, and flavone acyl-glycosides such as apigenin C-pentoside-C-hexoside-O-(5-hydroxy-feruroyl), also showed peak accumulation after 28 days of treatment with the bio stimulant, with a T/C ratios of 2.55, 2.47, and 2.55, respectively. In the leaves, apigenin appeared to be accumulated in its di- and tri-glycoside forms in the treated plants compared to the control, with the highest T/C ratio of 2.94 for apigenin C-pentoside-C-pentoside at D16 and 2.1 for apigenin C-pentoside-C-hexoside-O-hexoside at D9. An accumulation of flavone acyl-diglycosides such as apigenin C-pentoside-C-hexoside-O-feruloyl, apigenin C-pentoside-C-hexoside-O-sinapoyl, and apigenin C-pentoside-C-hexoside-O-(5-hydroxy-feruloyl), was also observed in the leaves of plants treated with the bio stimulant, compared to those of the control, at different developmental stages. These results indicate that the bio stimulant promotes the synthesis of flavonols and flavones in wheat plants. Buer et al. (2006) have shown that in *Arabidopsis thaliana*, *flavonoid*-deficient mutant plants display a delay in root gravitropism and growth compared to the wild-type plants, suggesting the role of flavonoids in root gravitropism and growth (Buer & Muday, 2004). Previous studies have shown that flavonoids, especially flavonols, regulate the transport of auxin, which has long been known as a key regulator of physiological processes, including root gravitropism and branching (Brown et al., 2001; Buer & Djordjevic, 2009; Buer et al., 2006; Peer et al., 2004). Taken together, we can hypothesize that treatment with the bio stimulant induces the biosynthesis of flavonoids that may act by modulating auxin movement in the root, which in turn improves roots gravitropism and growth, leading to the development of deep roots and thereby enhancing the resistance of wheat to lodging stress.

## Conclusions

Overall, our research provides strong evidence that metabolic fingerprinting is an effective tool for evaluating bio stimulants and assessing their impact on plants. Specifically, our results indicate that the application of Agro-K® elicited a metabolic response in wheat plants, resulting in variations in metabolite content associated with enhanced root growth and cell wall lignification, ultimately contributing to improved lodging resistance. These metabolomic results are consistent with the agronomic findings that contributed to the product registration (CE fertilizer—Bio stimulant, complies with regulation UE 2019 – 1009). Based on our findings, we suggest that HCAAs could serve as novel target molecules for evaluating the efficacy of commercial bio stimulants used in agriculture, particularly in improving lodging and abiotic stress resistance, since the accumulation of these metabolites in plants is associated with root development, soil anchorage, and cell wall stiffening and lignification. Further research to investigate the direct impact of treatment with the bio stimulant on the cell wall structure by measuring the *cell wall* thickness, as well as on the cell wall composition by analysis and quantification of lignin would be essential to corroborate our findings. However, our results pave the way for studying the effect of bio stimulation using metabolomic approaches. They also validate the feasibility of monitoring a systemic response to an elicitor over time and in fluctuating environments, such as directly in field conditions.

## Data Availability

The data presented in this study are available on request from the corresponding author.

## Declarations

### Sample availability

Samples of the compounds are available on request from the corresponding author.
